# Natural Colorants: Historical, Processing and Sustainable Prospects

**DOI:** 10.1007/s13659-017-0119-9

**Published:** 2017-01-16

**Authors:** Mohd Yusuf, Mohd Shabbir, Faqeer Mohammad

**Affiliations:** 1 Department of Chemistry, Y.M.D. College, Maharshi Dayanand University, Nuh, Haryana 122107 India; 2 Department of Chemistry, Jamia Millia Islamia (A Central University), New Delhi, 110025 India

**Keywords:** Natural colorants, Textiles, Sustainability, Processing, Adsorption, Application

## Abstract

**Abstract:**

With the public’s mature demand in recent times pressurized the textile industry for use of natural colorants, without any harmful effects on environment and aquatic ecosystem, and with more developed functionalities simultaneously. Advanced developments for the natural bio-resources and their sustainable use for multifunctional clothing are gaining pace now. Present review highlights historical overview of natural colorants, classification and predominantly processing of colorants from sources, application on textiles surfaces with the functionalities provided by them. Chemistry of natural colorants on textiles also discussed with relevance to adsorption isotherms and kinetic models for dyeing of textiles.

**Graphical Abstract:**

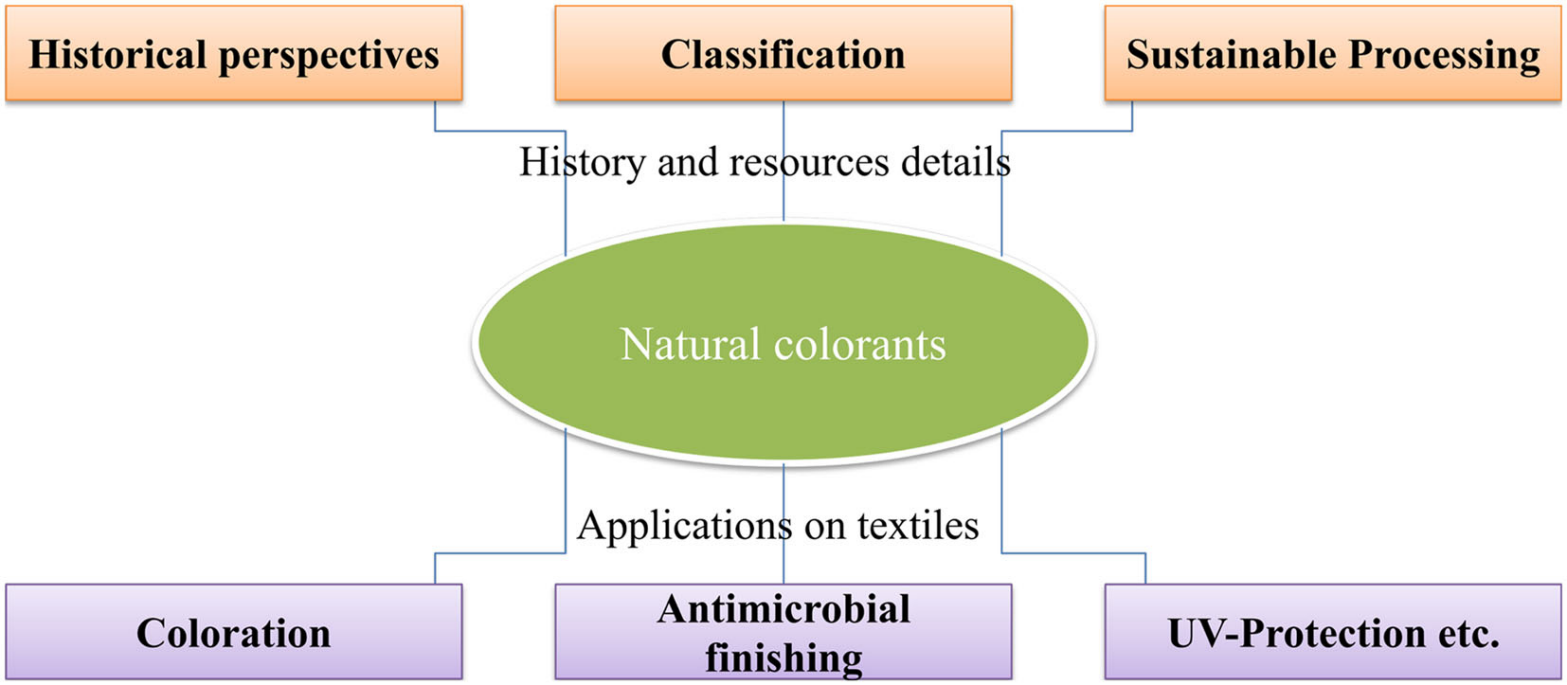

## Introduction

Nature has always dominated over synthetic or artificial, from the beginning of this world as nature was the only option for human being then, and now with advantageous characteristics of naturally derived materials over synthetics giving them priority. Color has always played an important role in the formation of different cultures of human being all over the world. It affects every moment of our lives, strongly influencing the clothes we wear, the furnishings in our homes. In the past, painters had used natural dyes extracted from plants, insects, molluscs and minerals for their paintings. The unique character of their works were the result of using different mixtures of dyes and mordants, as varnishes and lacquers responsible for cohesion of the pigments and protection of the layers destroyed by environmental effects. Natural dyes were also used in clothings, as well as in cosmetic industry (Henna, Catechu), pharmaceutical industry (Saffron, Rhubarb) and in food industry (Annatto, Curcumin and Cochineal) [1, 2]. As now public’s awareness for eco-preservation, eco-safety and health concerns, environmentally benign and non-toxic sustainability in bioresourced colorants, have created a revolution in textile research and development [3–7]. Also, environmental and aquatic preservation aspects forced Western countries to exploit their high technical skills in the advancements of textile materials for high quality, technical performances, and side by side development of cleaner production strategies for cost-effective value added textile products [8].

**Fig. 1:**
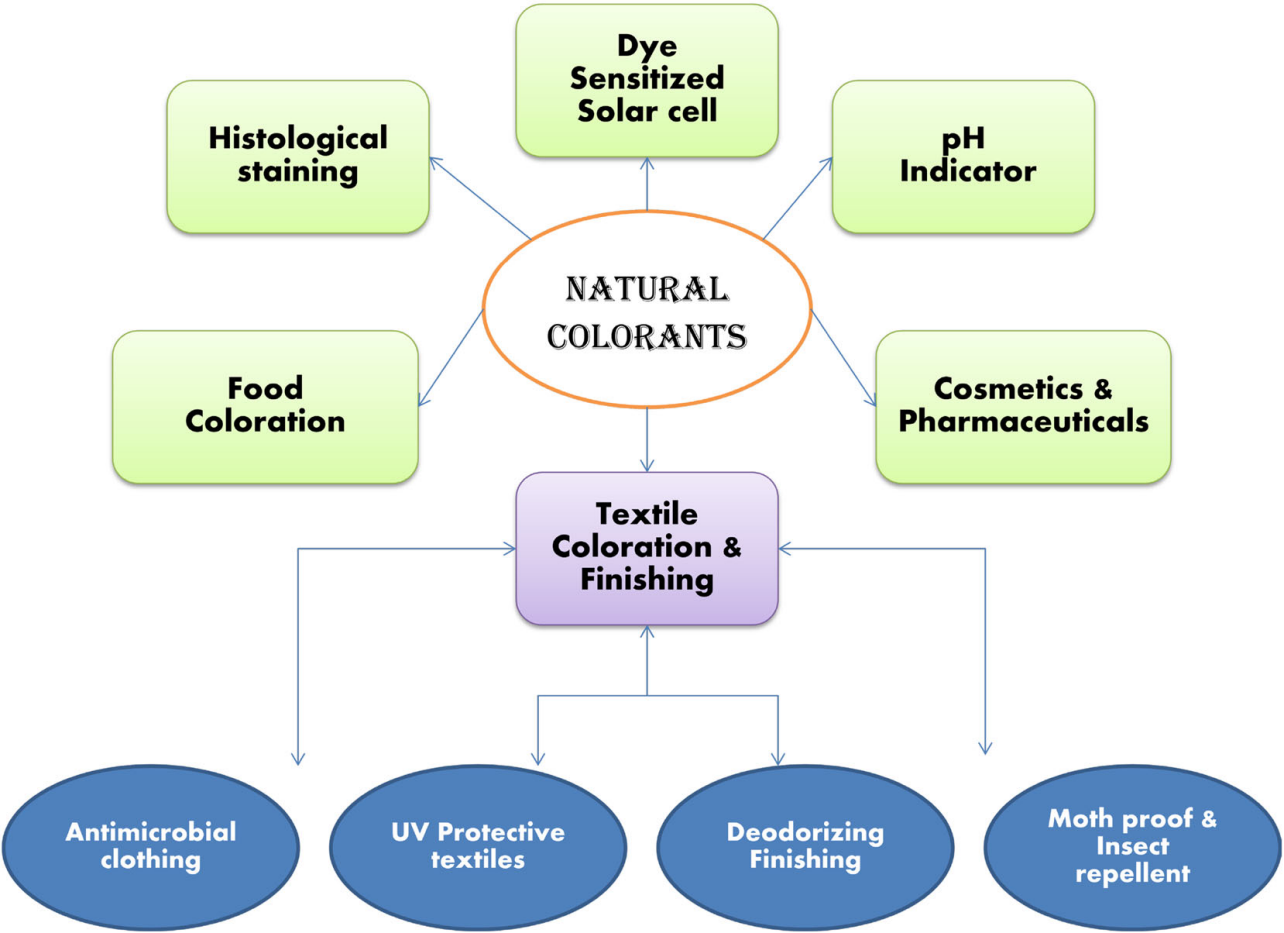
Applications of natural colorants

However, during last few decades, ecological concerns related to the use of most of the synthetic dyes, motivated R&D scholars all over the globe to explore new eco-friendly substitutes for minimizing their negative environmental impacts, and various aspects of bio-colorant applications (Fig. 1). Therefore, both qualitative and quantitative research investigations have been undertaken all over the world on colorants derived from cleaner bio-resources having minimal ecological negative impacts [9–13]. Consequently, strict Environmental and Ecological Legislations have been imposed by many countries including Germany, European Union, USA and India [14]. As a result, eco-friendly non-toxic naturally occurring bio-colorants have gaining re-emergence as a subsequent alternative through green chemistry approaches with wide spread applicability to textile coloration and other biomedical aspects [15]. This review article is intended to discuss the isolated and dispersed impacts of bio-colorants derived from bio-resources, via significant aspects including, classification, extraction and dyeing, sustainability, adsorption and chemical kinetics and recent technological applications with future prospects.

## Historical Background and Classification

The archaeological textile research involves the investigation through scientific technologies to detect the chemical composition and, to identify the sources of the dyestuffs used in old textiles. These studies of the colorants used by ancient peoples include a multidisciplinary research, combines micro-analytical chemistry, spectroscopical methods, history, archaeology, botany etc. The dyestuffs applied onto textile materials past civilizations have been examined to investigate the development and technological advancements in textile dyeing through various archaeological periods. In the past decades, researchers are very much benefited from the instrumental analyses of ancient artifacts and colorants were analyzed with micro chemical tests, such as TLC, HPLC, reversed phase HPLC, FT-IR spectroscopy, UV–Visible spectroscopy, X-ray fluorescence, and energy dispersive X-ray (EDX) spectroscopic techniques [16–19]. Consequently, some more influencing surface micro-analytical techniques, such as X-ray photoelectron spectroscopy (XPS), mass spectroscopy (MS), high performance mass spectroscopy (HPMS), time-of-flight secondary ion mass spectrometry (ToF–SIMS) and atomic emission spectroscopy (AES) have been employed to study ancient materials of art and archaeology, which provided the widest range of information with the minimal degree of damage to the tested object [20–24].

In the Ancient Stone Age, descriptions have shown that peoples were used various powders made up of colored minerals, and applied to their hair and body parts to confer magic powers while hunting as well as occasional dressings. Many antiquity writers regarded the Phoenicians as the pioneers of purple dyeing and they attribute the beginning of this art to the maritime occasion city of Tyre in the year 1439 BC. For this purpose they had used murex shells. Also, ancient purple dyeing craft in the Roman Empire was reported and, prove the cultural importance of natural colors, the techniques of producing and applying dyes. The spectroscopic analysis of ancient Egyptian cuneiform texts have found dyed with bio-colorants which was traded by the ingenious and industrious craftsman, like madder, Murex sp., Tyrian purple, *Indigofera* sp. etc. [25, 26]. Ancient North African dyers were used bio-colorants derived from madder (*Rubia tinctoria*), cochineal (*Dactylopius coccus*) and kermes (*Kermes vermilio*) as sources of dyes and pigment lakes, but they were much more affordable and were widely used for dyeing and in medieval miniature paintings as well as in cosmetics [27, 28]. The Egyptians were conscious as they excelled in weaving for many inscriptions extol the garments of the gods and the bandages for the dead, principally dyed with archil, a purple color derived from certain marine algae found on rocks in the Mediterranean Sea; alkanet, a red color prepared from the root of *Alkanna tinctoria*, *Rubia tinctorum*, which generates red colored materials, woad (*Isatis tinctoria*), a blue color obtained by a process of fermentation from the leaves, and indigo from the leaves of the *Indigofera* species [29–31].

Natural originated bio-colorants have been discovered through the ingenuity and persistence of our ancestors, for centuries and may be found veiled in such diverse places as the plant roots (i.e. *Rubia tinctorum*), rhizomes (*Rheum emodi, Curcuma longa*), insects (*Lacifer lacca,* Kermes) and the secretions of sea snails. However, in Mediterranean civilization, the most valuable colors were indigo for the blues, madder for the reds and 6,6′-dibromoindigo for purple [2, 32]. Human being has always been interested in colors; the art of dyeing has a long history and many of the dyes go back to pre-historic days. The nails of Egyptian Mummies were dyed with the leaves of henna, *Lawsonia inermis* [33, 34].

Chemical tests of red fabrics found in the tomb of King Tutankhamen in Egypt show the presence of alizarin, a pigment extracted from madder. Kermes (*Coccus ilicis/Kermes vermillio*) which flourished on evergreen Oak (*Quercus coccifera*) in Spain, Portugal and Morocco is identified in the Book of Exodus in the Bible, where references are made to scarlet colored linen. Sappan wood was exported from India to China as early as 900 BC [35–37]. The relics from excavation at Mohanjodaro and Harappa (Indus Valley Civilization), Ajanta Caves Painting and Mughal dyeing, printing and painting, show the use of natural dyes such as Madder, Indigo and Henna. Excavation at Mohanjodaro shows the use of madder on cotton clothes is the testimony of genius Indian craftspersons. Classics like Mahabharata and Code of Manu, refer to the colored fabrics, endowing them with specific social & religious connotations [38]. Colors communicate emotions with greater clarity; they were not used randomly but reflected the mood and emotions of the occasion. Irrespective of religious differences red became the symbol of bride’s suhag, saffron the color of earth, yellow the color of spring, black is associated with mourning and white with widowhood, representing life bereft of happiness [39].

The most famous and highly prized color through the ages was Tyrian purple, noted in the Bible, a dye obtained from the hypobranchial glands of several marine gastropods molluscs of the genera *Murex, Bolinus, Purpura, Plicopurpura* and *Thias* and it is probably the most expensive dye in the history of mankind. Indian dyers were perfect in the process of bleaching, mordanting and dyeing by the fourth and fifth century AD. Records of compound colors of black, purple, red, blue and green with various shades of pink and gold are available in contemporary accounts of tenth century, amongst them, the anonymous; Hudud-ul-Alam (982–983) is most important document in the history of dyeing. In the period of Mughal reign (1556–1803) dyers used Madder, Myrobalan, Pomegranate, Turmeric, Kachnar, Tun, Dhao, Indigo, Henna, Catechu, Saffron and Patang as natural dyes and pigments and the mordants which were used in those days were soluble salts of Aluminium, Chromium, Iron and Tin which adheres strongly with fibres and give fast colors [32, 40, 41]. Mordanting and block printing techniques are said to be originated as pre-historic antiquity of India and major towns like Delhi, Farrukhabad and Lucknow were the famous towns of Mughal era as stated in Mrs. Hameeda Khatoon Naqvi’s article *Dyeing of cotton goods in the Mughal Hindustan* (1556–1803) [42].

### Classification of Natural Colorants

**Fig. 2:**
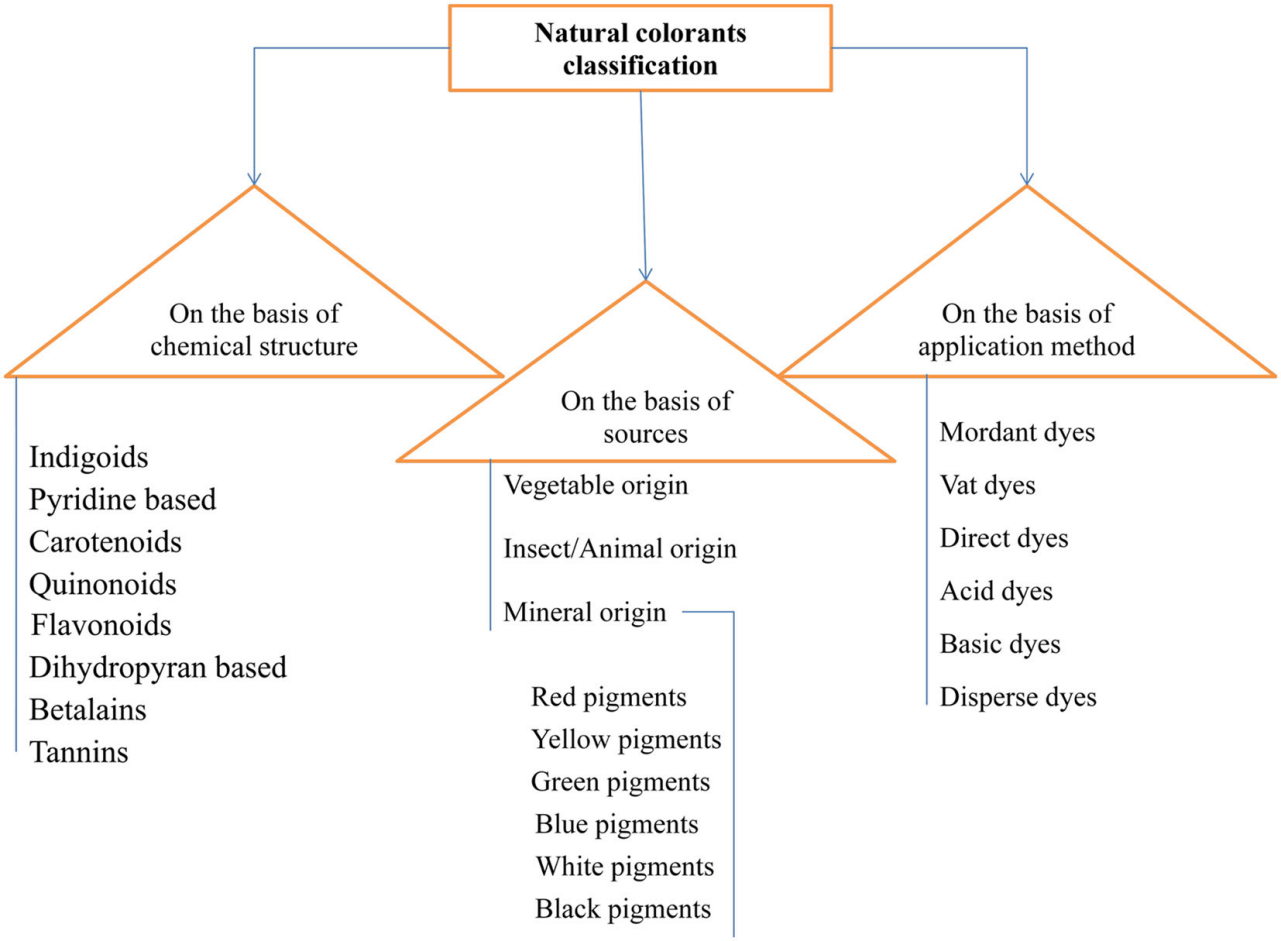
Classification chart for natural colorants

Natural dyes have been classified in a number of ways (Fig. 2). Major basis of classification of natural dyes are their production sources, application methods of them on textiles and their chemical structure.

#### Based on Chemical Structure

Classification of natural dyes on the basis of chemical structure is the most appropriate and widely accepted system of classification, because it readily identifies dyes belonging to a particular chemical group which has certain characteristic properties (Table 1).

**Table 1:** Classification based on chemical structure with typical examples [13, 41, 44, 57, 58, 60, 64, 67, 96]

Classes	Chemical structures
Indigoids	
Pyridine based	
Carotenoids Acyclic Cyclic	
Quinonoids	
Flavonoids	
Dihydropyran based	
Betalains	
Tannins	

##### Indigoids [43–46]

Indigoids (Indigo and Tyrian purple) are perhaps the most important group of natural dyes and the oldest dyes used by human civilizations. Natural indigo is a dye having distinctive blue color with long history and is regarded as one of the most important and valuable of all coloring matters. Indigo is extracted from *Indigofera* spp. (*Indigofera tinctoria*), *Polygonam tinctorium* (dyer’s knotweed), *Perisicaria tinctoria,* and *Isatis tinctoria* (woad) [47]. But nowadays large percentage of indigo (Several thousand tons per year) is synthetic. The dye Tyrian purple (C.I. 75800) also known as Tyrian red, royal purple and imperial purple is a bromine-containing reddish-purple natural dye, derived from the hypobranchial glands of several marine predatory sea snails in the family *Muricidae*. This dye has excellent light fastness properties [48].

##### Pyridine Based Dyes

Berberine **(**natural yellow 18; C.I. 75160), an isoquinoline alkaloid with a bright yellow color, is the only natural dye belonging to this class [49]. Some important berberine yielding dye plants are *Berberis aristata*, *Berberis vulgaris* [50], *Phellodendron amurense* [51], and *Rhizoma coptidis* [52].

##### Carotenoids [53, 54]

Carotenoids also called tetraterpenoids are brightly colored natural organic pigments found in the chloroplast and chromoplast nearly in all families of plants and some other photosynthetic organisms. Only plants, fungi and prokaryotes are able to synthesize carotenoids [55]. The color of the carotenoids is due to the presence of long conjugated double bonds. They absorb light in the 400–500 nm region of the spectrum and this give rise to yellow, orange and red color [56]. *Bixa orellana, Crocus sativus*, *Curcuma longa, Nyctanthes arbor*-*tristis*, and *Cedrela toona,* are some of carotenoids source plants.

##### Quinonoids [57, 58]

Quinonoids are widely distributed and occurs in large numbers in nature ranging from yellow to red. Chemical structures of naturally occurring quinones are more diverse than any other group of plant pigments. On the basis of chemical structure these dyes are further classified as benzoquinones, α-naphthoquinones and anthraquinones. *Carthamus tinctorius* (Safflower), *Choloraphora tinctoria* (Gaudich), *Lawsonia inermis/Lawsonia alba* (Henna/Mehendi), *Juglans regia* (Walnut), *Plumbago capencis* (Chitraka/Chita), *Drosera whittakeri* (Sundew), *Tabebuia avellanedae* (Taigu/Lapachol), *Alkanna tinctoria* (Ratanjot/Alkanet), *Lithospermum erythrorhizon* (Tokyo Violet/Shikone), *Dactylopius coccus* (Cochineal*), Kermes vermilio/Coccus ilicis, Laccifer lacca/Kerria lacca/Coccus lacca, Rubia tinctorum, Rubia cordifolia* (Indian Madder), *Rheum emodi* (Himalayan rhubarb), *Oldenlandia umbellata* (Chay Root), and *Morinda citrifolia* (Al/surangi/ach) are the natural resources for quinonoids class; subclass anthraquinonoids and naphthoquinonoids [6, 7, 13, 43, 59].

##### Flavonoids [60]

Flavonoids provide the largest group of plant dyes ranging in colors from pale yellow (isoflavones) through deep yellow (chalcones, flavones, flavonols, aurones), orange (aurones) to reds and blues (anthocyanins). Various plant sources of flavonoid dyes [61–65] are *Reseda luteola* (Weld), *Allium cepa* (Onion), *Artocarpus heterophyllus/Artocarpus integrifolia* (Jackfruit), *Myrica esculenta* (Kaiphal), *Datisca cannabina* (Hemp), *Delphinium zalil* (Yellow Larksur), *Gossypium herbaceum*, *Sophora japonica/Styphnolobium japonicum*, *Butea monosperma/Butea frondosa* (Flame of the forest/Palas), *Mallotus philippinensis* (Kamala), *Bignonia chica/Arrabidaea china* (Carajuru/Puca), *Commelina communis, and Pterocarpus santalinus* (Red Sandalwood).

##### Dihydropyran Based Dyes

These pigments comprise of brazilin (C.I. 75280) from brazilwood (*Caesalpinia sappan*) and haematoxylin (C.I. 75290) from logwood (*Haematoxylon campechianum)*.

##### Betalains

Betailains are a class of water soluble nitrogen containing plant pigments of the order Caryophyllales which comparise of the yellow betaxanthins and the violet betacyanins. *Opuntia lasiacantha* [66] and *Beta vulgaris* (Beetroot) are common natural sources for betalains class of colorants [67].

##### Tannins

Tannins are astringent vegetable products found in most of the vegetable kingdom. Tannins are obtained from the various parts of the plants such as fruit, pods, plant galls, leaves, bark, wood, and roots. Tannins are defined as, water soluble phenolic compounds having molecular weights between 500 and 3000. Tannins are usually classified into two groups-hydrolysable (pyrogallol) and condensed tannins (proanthocyanidins). The hydrolysable tannins are polyesters of a sugar moiety and organic acids, grouped as gallotannis and ellagitannins which on hydrolysis yield galllic acid and ellagic acid, respectively [3, 68].

Tannins are primarily used in the preservation of leather. Tannins are used in glues, inks, stains and mordants. Tannins are also used for heavy metal removal in surface water treatment. Tannins play very important role in dyeing with natural dyes by improving the affinity of fibres towards different dyes. By mixing with different natural dyes it gives different shades like yellow, brown, grey and black. *Acacia catechu* (Cutch), *Terminalia chebula* (Harda), *Punica granatum* (Pomegranate/Anar), *Quercus infectoria* (Gallnut), are plant sources for tannins [3, 41, 65, 68, 69].

#### Based on Production Sources [70, 71]

On the basis of origin, natural dyes can be classified into three classes:

##### Vegetable/Plant Origin

Most of the natural dyes belong to this category. The colorants derived from various plant parts such as flowers, fruits, seeds, leaves, barks, trunks, roots, etc. fall in this category. In India there are nearly four hundred fifty dye yielding plants.

##### Insect/Animal Origin

Red animal dyes obtained from exudation of dried bodies of insects namely, Cochineal, Kermes, *Laccifer lacca/Kerria lacca* and molluscs such as carminic acid (cochineal), kermesic acid (Kermes), laccaic acid (Lac dye), and Tyrian purple belong to this category. They are well known for dyeing purposes from ancient times.

Natural colorants obtained from plants and animals are discussed in detail later in chemical structure basis classification with examples.

##### Mineral Origin

Various pigments from inorganic metal salts and metal oxides belong to this category of natural dyes. The most important mineral pigments are as follows:Natural colorants from mineral origin can further be classified with their colors.

*Red Pigments* Cinnabar, Red Ochre, Red lead and Realgar are some of the examples of red pigments originate from minerals. Cinnabar, also known as vermillion, refers to common bright scarlet to brick-red form of mercury sulphide (HgS), a common source ore for refining elemental mercury and serves directly as dyeing pigment. Red Ochre (Geru in Hindi) is a natural earth pigment containing anhydrous and hydrated iron oxide (Fe_2_O_3_·nH_2_O). The color of red ochre is not as bright as that of Cinnabar but it is found in several hues, which ranges from yellow to deep orange or brown. Red Ochre is very stable compound and is not affected by light, acids and alkalies. Fine red ochre is obtained by washing its crude variety. Red ochre is used by monks to color their robes. Red lead (Sindur in Hindi) (Pb_3_O_4_ or 2[PbO]·[PbO_2_]) is a bright red or orange crystalline or amorphous pigment has been used in Indian paintings in abundance. Realgar (α-As_4_S_4_) (Manasila in Hindi) is an arsenic sulphide mineral commonly known as Ruby sulphur or ruby of arsenic, found in combination with orpiment (As_2_S_3_) which is also a mineral of arsenic. Both are sulphides of arsenic but these are not safe and have not been used much in paintings.

*Yellow Pigments* Yellow Ochre (Ram Raj), Raw Sienna, Orpiment and Litharge (Massicot) are classified in yellow pigments due to their yellow color range. The color of the yellow ochre is on the account of the presence of various hydrated forms of iron oxide, particularly the mineral limonite (Fe_2_O_3_·H_2_O). The pigment is prepared from natural earth by selection, grinding, washing, and lavigation. Raw sienna belongs to Sienna (Siena earth) class of earth pigments containing iron oxide and manganese oxide. Along with ochre and umber it is first pigment to be used in human cave paintings. It is considerably transparent and used in paintings as a glaze for its transparency. Orpiment (Hartal in Hindi) is a deep orange-yellow colored arsenic sulphide mineral and gives a brilliant rich lemon-yellow color. Chemically, it is yellow sulphide of arsenic (As_2_S_3_). Besides being used as a pigment, it has been used to tint paper to make it yellow. This process also imparts an insecticidal property to paper. Litharge (Massicot) is natural secondary mineral forms of lead oxide (galena) and is made by gently roasting white lead. White lead, which is chemically lead carbonate (2PbCO_3_·Pb(OH)_2_), upon decarboxylation and dehydration gives on heating at a temperature of about 300 °C is converted into a pale yellow powder which is monoxide of lead (PbO).

*Green Pigments* Terre-Verte (Green Earth), Malachite and Vedgiris are examples of green pigments. Among them, terre-verte has been the most widely used since earlier times. Green earth is a mixture of hydrosilicates of Fe, Mg, Al, and K (gluconite and celadenite) but other minerals are likely to be present. The color of green earth, depending on the source, varies from place to place. The hues are from yellow green to greenish grey and are not affected by light or chemicals. Malachite is a copper carbonate hydroxide mineral with chemical formula of Cu_2_(OH)_2_CO_3_. This opaque, green banded mineral crystallizes in the monoclinic crystal system. Vedgiris was a common pigment used in paintings during Mugal era and later in miniature paintings. It is the normal acetate of copper [Cu(CH_3_COO)_2_] and is prepared by the action of vinegar on copper foils. The pigment obtained is very bright and deep green. However, it has disadvantage that it chars the paper or textile if not used carefully.

*Blue Pigments* Ultramarine Blue and Azurite are blue pigments. Ultramarine blue (Lajward in Hindi) is a deep blue colored pigment obtained from the mineral lapis lazuli, which is semi-precious stone. It has been used in miniature paintings in India. Lapis lazuli was imported to India from Afghanistan during fourteenth and fifteenth centuries. Azurite [Cu_3_(CO_3_)_2_(OH)_2_] is a soft, deep, blue colored pigment produced by weathering of copper ore deposits. This pigment was extensively used in Chinese paintings but rarely in Indian paintings. However, it has been reported that this mineral is found along with Indian copper ores.

*White Pigments* Chalk (White Lime), White lead and Zinc White. Chalk is one of the forms of calcium carbonate (CaCO_3_). It has been extensively used in paintings. Chalk is found with limestone deposits and has been used as pigment from very early times. In India, conch shell white was favoured by artists and is believed to have special properties. White lead (PbCO_3_) is a complex salt containing both carbonate and hydroxide. It was formerly used as an ingredient in lead paint. It occurs in nature as the mineral Cerussite. However, normally white lead is prepared artificially. Zinc white (ZnO) (Safeda in Hindi) is another important pigment used in painting. Archaeological evidence dates back to the use of zinc white as pigments in India before it was introduced in Europe. Other white pigments are Talc, Barium White and Titanium White. Titanium White is titanium dioxide (TiO_2_), used in textiles as delustrants.

*Black Pigments* Charcoal Black, Lamp Black, Ivory Black, Bone Black, Graphite, Black Chalk and Terre-noire (Black Earth) are among the list of black pigments. Well ground charcoal has often been used as black pigment. In India, charcoal prepared from twigs and woods of tamarind tree after burning in a closed pot, is powdered to make black pigment. Some other substances which after charring were used for preparing black pigment are the shells of almonds and coconuts. The charcoal so produced is soft and gives homogeneous and fine black pigment. By far, the most important black used India is ‘Kajal’ prepared by burning oil in a lamp and depositing the soot on an earthen bowl. Ivory black is prepared by charring ivory cuttings in a closed earthen pot and then grinding, washing and drying black residue. The black so prepared is very intense. It is not favoured now for ecological and animal rights considerations. Bone black is prepared by charring animal bones in closed earthen pots. It is not as intense as ivory black but used as a substitute. Powdered graphite, a mineral found in different parts of India, has been used as writing material. It gives a dull grey pigment. However, it has mostly been used for drawing rather than for painting. Black chalk is the name given to black clay used for paintings and terracotta. Terre-noire is the same as black clay. It is a mixture of carbonate of calcium, iron and manganese with clay.

#### Based on Application Methods

Based on method of application, natural dyes have been classified into following classes:

##### Mordant Dyes

Mordant dye/colorants are those which can be bound to a material for which it otherwise has little or no affinity by the addition of a mordant, a chemical that increases the interaction between dye and fibre. This classical definition of mordant dyes has been extended to cover all those dyes which are capable of forming complex with the metal mordant. Most of these dyes yield different shades or colors with different mordants with different hue and tone.

##### Vat Dyes

Vat dyeing is a process that takes place in bucket or vat. They are insoluble in their colored form, however can undergo reduction into soluble colorless (leuco) form which has an affinity for fibre or textile to be dyed. Re-oxidation of the vat dyes converts them again into ‘insoluble form’ with retention of original color. Only three natural dyes belong to vat dyes: indigo, woad and tyrian purple.

##### Direct Dyes

Direct dyes are water-soluble organic molecules which can be applied as such to cellulosic fibres such as cotton, since they have affinity and taken up directly. Direct dyes are easily applied and yield bright colors. However, due to the nature of chemical interaction, their wash fastness is poor, although this can be improved by special after-treatment. Some prominent examples of direct natural dyes are turmeric, annatto, harda, pomegranate and safflower.

##### Acid Dyes

Acid dyes are also another type of direct dyes for polyamide fibres like wool, silk and nylon. These dyes are applied in acidic medium and they have either sulphonic acid or carboxylic acid groups in the dye molecules. At least one natural dye, saffron has been classified as acid dye. This dye has two carboxylic acid groups.

##### Basic Dyes

Basic dyes are also known as cationic dyes. These dyes on ionization give colored cations which form an electrovalent bond with the carboxyl group of wool and silk fibres. These dyes are applied from neutral to mild acidic condition. Berberine has been classified as basic dye. Structurally, this dye carries a non localized positive charge which resonates in the structure of the dye, resulting in poor light fastness.

##### Disperse Dyes

Disperse dyes are water insoluble dyes which dye polyester and acetate fibres. The principle of disperse dyeing is recent one as compared to the age of natural dyeing. However, in view of their structural resemblance and solubility characteristics it is felt that some of the natural dyes such as lawsone, juglone, lapachol and shikonin can be classified as disperse dyes.

## Processing and Sustainability Aspects

### Extraction

**Fig. 3:**
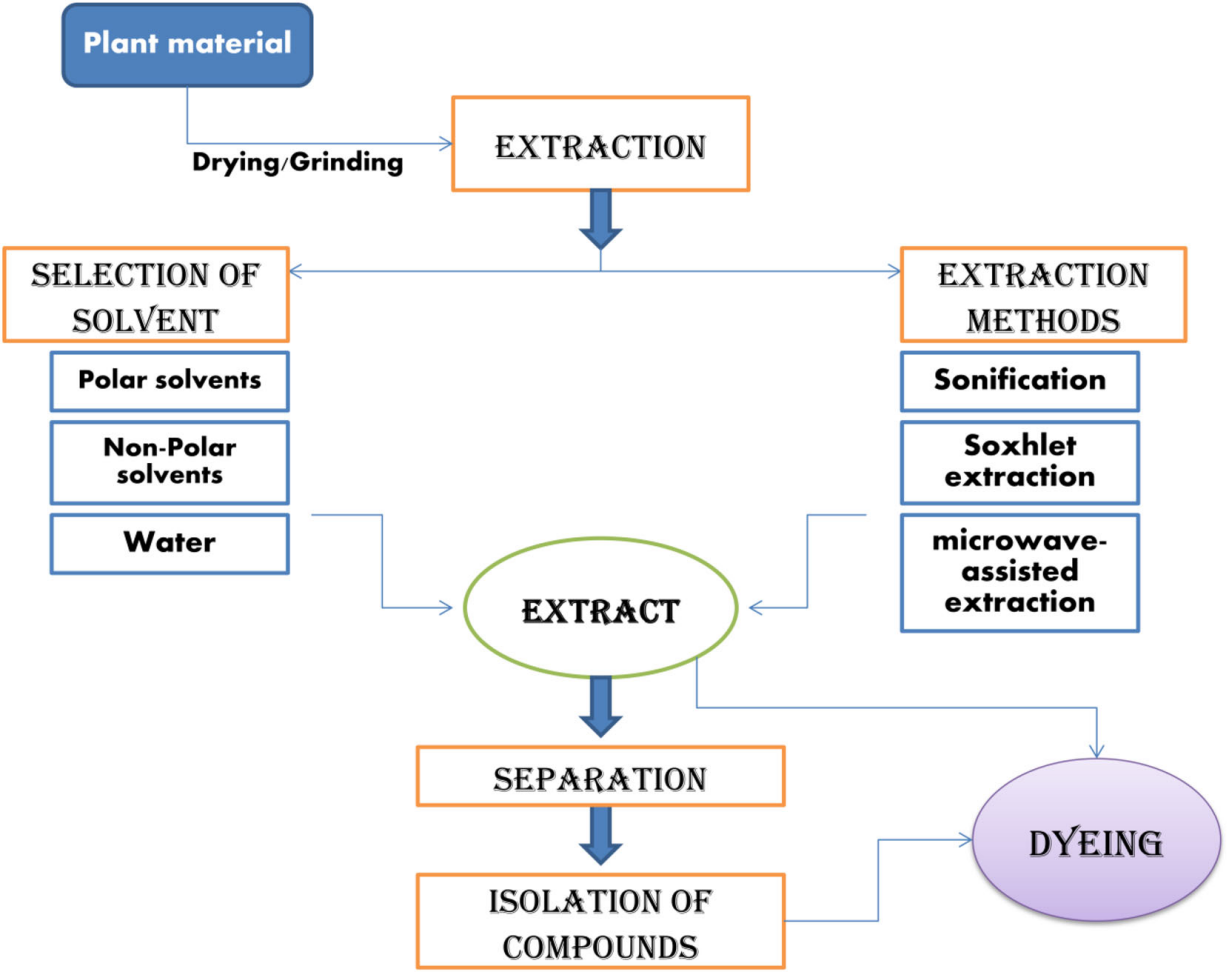
Schematic representation for extraction of natural colorants

**Fig. 4:**
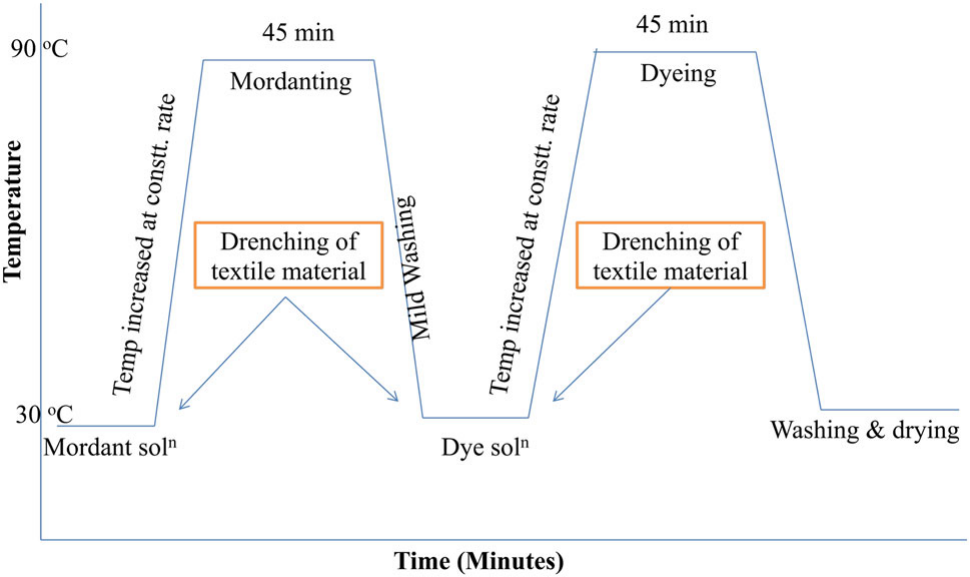
Schematic representation for mordanting and dyeing profile

Natural colorants classified in the previous section, are to be extracted from their sources to be applied on textiles. Various techniques, solvents and parameters were used for extraction in natural dyeing literature. Figures 3 and 4 represent the schematic representation for extraction of natural colorants and mordanting and dyeing profile, respectively.

First step of extraction is preparation of the plant material ready to be extracted such as collection of plant materials, drying and grinding to make homogenous mixture and to enhance surface area for maximum contact to solvent used. After that most important step, is selection of solvent, depending on the nature of compounds to be isolated or extracted. To extract hydrophilic compounds polar solvents such as methanol, ethanol or ethyl-acetate can be used and for extraction of lipophilic compounds, dichloromethane or a mixture of dichloromethane/methanol in ratio of 1:1 can be used. Various methods, including sonification, heating under reflux, soxhlet extraction and others commonly used depending on the target compound’s polarity and thermal stability. Some modern methods are also used for extraction like solid-phase micro-extraction, supercritical-fluid extraction, pressurized-liquid extraction, microwave-assisted extraction, solid-phase extraction and surfactant-mediated techniques owing to their advantages in terms of yield and easy collection of extracts. Extraction obtained generally is mixtures of compounds which are further to be separated by separation techniques named some of them are adsorption chromatography, thin layer chromatography (TLC) and high performance liquid chromatography (HPLC). Then compounds are to be characterized by spectroscopic techniques such as ultra-violet spectroscopy (UV), fourier-transform infrared spectroscopy (FTIR) etc. [72, 73].

Although, much research have been explored in the past with extraction of colorants from plant sources. A process has been used which employs sulfur dioxide for extraction in a patent [74]. The extract is passed through an ion exchange column to absorb the anthocyanin material and the adsorbed material is eluted by means of acetone, alkali or dimethyl formamide (DMF). Moreover, a process for the extraction of carotenoid dyes from pre-dried natural starting materials is described in a patent in 1998 using compressed gases such as propane and/or butane in which organic entraining agents can be additionally added in order to facilitate and complete the extraction process. With the aid of this process highly concentrated carotenoid dyes are obtained in high yield [75]. Extraction of anthocyanin dyes from red grape pomace with carbon dioxide, along with other solvents either methanol or water at high pressures were studied by Mantell et al., Various extraction parameters such as temperature, pressure, solvent flow rate, co-solvent percentage, solvent type and extraction time were studied for optimized results and the quantification was performed by colorimetric method. 20 mol% of methanol, 100 bar pressure, 60 °C temperature and 22 mmol/min flow rate were found optimized parameters for maximum yield [76]. Bechtold et al., extracted anthocyanin dyes from red pomace for textile dyeing in distilled water 1;20 of material to liquor ratio (M:L) at 95 °C temperature for 60 min [8]. Crude dyestuff from pomegranate peel for dyeing was extracted with 1:5 of material to solvent (ethanol water) ratio for 60 min at 60 °C of temperature. Obtained filtrate was distillated for 3 h at 70 °C temperature in soxhlet apparatus and concentrated dye was obtained for dyeing [9]. Dye (mixture of gallic acid, ellagic acid, quercetin and rutin compounds) was extracted from fresh eucalyptus leaves, dried in sunlight for 1 month and crumbled using a blender, by the reflux technique; 70 g of crumbled eucalyptus leaves was mixed in a litre of distilled water and refluxed for 1 h. Filtrate obtained by filtration was evaporated under reduced pressure and dried and used for dyeing silk and wool [77]. Aqueous extraction of tannin colorants from tea was prepared by adding 2 and 5 g commercially available tea powder to 100 ml distilled water and the mixture was stirred, heated, held at the boil for 30 min, allowed to stand for 15 min and then filtered and used for dyeing cotton [78]. Anthraquinone dyes were extracted from *Cassia tora* L. seed using various pH buffer solutions (pH 2–11) for 3 days at room temperature in material-liquor ratio of 1:10. The *Cassia tora* L. extract solution (natural dye solution) obtained at pH of buffer 9 was found of highest K/S and a yellowish red solution for dyeing of cotton and silk [79]. Coffee sludges were also used to extract the yellow colorant from them. Water was used as extractant at 90 °C for 90 min in material-liquor ratio of 1:10. The obtained dye solution was used for dyeing of cotton, wool and silk, and colorimetric, fastness and deodorising properties were evaluated [80].

### Mordanting and Dyeing

Today, a large number of researchers around the globe are working on natural colorants advancements. After extraction processing, next step is application of natural colorants on textiles with or without the help of mordants. From the start of their use for textile dyeing via conventional methods to innovative and advanced methods trending in recent times, natural colorants are gaining their space in textile coloration and functionalization.

#### Mordanting Methods

To get the highest substantivity of natural colorants towards textiles, some metal salts or other chemicals or compounds, so called mordants are used with colorants. Mordanting is classified on the basis of application time of mordants that are pre-, meta- and post-mordanting.

##### Pre-mordanting

Textile materials treatment with mordants prior to dyeing is called as pre-mordanting, which provides exclusive, sufficient time and sites on textile material to bind to the mordants. A proper layering of dye, mordant and textile material formed in this type of processing of natural colorants on textiles. Metal complexation with textile surface sites from one side and from dye on the other make the color fast to light, washing and rubbing. Chelating complexation of this processing makes the proper energy dissipation of photons of light in the complex and provide better light fastness to dyed materials. Optimum utilization of resources in pre-mordanting makes this more sustainable towards environment and flora and fauna.

##### Meta-mordanting/Simultaneous Mordanting

Both mordants and dyes are dissolved into the dye bath simultaneously for dyeing. This kind of processing makes a large wastage of the resources, both dye and mordant, by complexation between each other. Some sites of the textile materials are occupied with mordants and some directly with the dye compounds causes to uneven dyeing. Three type of complexation occurs that are between textiles and mordants, textiles and dyes, and between dyes and mordants leads to overloading of dye effluent into the ecosystem, a threat to sustainability issues.

##### Post-mordanting

In this method, dye material or colorants are applied first to the bare textile material and then mordanting is carried out. This processing mainly applied to broaden the shade range with mordant complexation with dye molecules over the surface of textile materials. This method may not be an appropriate to fasten the color fastness.

In a general way, metallic mordants can be categorized as, (a) conventional mordants that are used from earliest times and (b) novel mordants that are used after the conventional mordants; newly invented as shown in Table 2.

**Table 2:** Several mordanting agents [1, 41, 43, 58]

Name of mordants	Chemical formula
Conventional mordants	
Alums	
Ammonia alum	Al_2_(NH_4_)_2_(SO_4_)_4_·24H_2_O
Chrome alum	Cr_2_K_2_(SO_4_)_4_·24H_2_O
Potash alum	Al_2_K_2_(SO_4_)_4_·24H_2_O
Soda alum	Al_2_Na_2_ (SO_4_)_4_·24H_2_O
Potassium dichromate	K_2_Cr_2_O_7_
Iron sulphate	FeSO_4_
Copper sulphate	CuSO_4_
Stannous chloride	SnCl_2_
Manganese chloride	MnCl_2_
Newly invented mordants	
Stannic chloride	SnCl_4_
Stannous sulphate	SnSO_4_
Calcium chloride	CaCl_2_
Calcium sulphate	CaSO_4_
Calcium hydroxide	Ca(OH)_2_
Magnesium sulphate	MgSO_4_
Aluminium sulphate	Al_2_(SO_4_)_3_
Aluminium chloride	AlCl_3_
Aluminium nitrate	Al(NO_3_)_3_
Copper acetate	(CH_3_COO)_2_Cu
Cuprous chloride	Cu_2_Cl_2_
Zinc tetrafluoroborate	Zn(BF_4_)_2_
Lanthanum oxide	La_2_O_3_
Chromium sulphate	CrSO_4_
Cobalt nitrate	Co(NO_3_)_2_
Ferrous chloride	FeCl_2_
Ferric chloride	FeCl_3_
Zinc sulphate	ZnSO_4_
Zinc chloride	ZnCl_2_
Nickel sulphate	NiSO_4_
Rhenium trichloride	ReCl_3_·6H_2_O
Neodymium trichloride	NdCl_3_·6H_2_O
Zirconium oxychloride	ZrOCl_2_·8H_2_O

#### Dyeing Methods

##### Conventional Dyeing System

From the time, textile dyeing started in past carried out conventionally. Textiles were directly processed with the dye bath at high temperatures. Numerous developments in dyeing context are observed in recent decades such as evaluation of effective mordants, printing techniques and dyeing procedures [34–36]. Several patents described the dyeing of textiles with indigo dye, first pre-treatment of textile materials with ecofriendly mordants and then with reduced indigo dye in inert atmosphere and then oxidation via flooding of cold water over the surface [81]. Cellulosic textile materials can be dyed with disperse dyes from supercritical CO_2_ by treating the textile materials with an auxiliary that promotes dye uptake, typically polyethylene glycol [82]. Coloration method for textiles using chemically formed gels with considerable freedom for making color designs and precise pattern prints, and can be used with conventional dyeing and printing equi pment was developed [83]. With the time, dyeing also matured with the development of optimization of dyeing parameters, and in recent times advanced technologies evolved like plasma treatment and enzymatic processing etc.

##### Advanced Dyeing Systems

Advanced technologies or methods are trending in dyeing in recent times owing to their improved results over the conventional dyeing. Plasma treatment and ultrasonic dyeing methods are modern, advanced and sustainability compatible methods used in technologically evolved textile industry. Plasma, also known as fourth state of matter and ultrasound waves is having sufficient energy responsible to affect the energy of dye bath components. Improved results in ultrasound-assisted dyeing are generally attributed to cavitation phenomena and other mechanical effects are produced such as dispersion (breaking up of aggregates with high relative molecular mass), degassing (expulsion of dissolved or entrapped air from fiber capillaries), diffusion (accelerating the rate of diffusion of dye inside the fiber) and intense agitation of the liquid. The acceleration in dyeing rates observed by many workers might be the cumulative effects of all these factors [84].

**Radiation Treatments (UV, Gamma Radiations and Plasma)**: Ultrasonic is also found effective in extraction of colorants. Ultrasonic power appreciably increased the color strength values of lac dye on textile material in comparison to conventional heating [85]. In case of *Eclipta* as natural dye on cotton fabric using both conventional and sonicator methods, higher color strength values obtained by ultra-sonication method. Dyeing kinetics of cotton fabrics were compared for both the methods and the time/dye uptake revealed the enhanced dye uptake showing sonication efficiency [86]. Higher extraction from red calico leaves, color strength and color fastness properties of gamma radiations particularly, 15 kGy dose treated cotton fabric were obtained by inducing surface modification [87]. In another study, dyeing was performed using un-irradiated and irradiated cotton with the extracts of un-irradiated and irradiated turmeric powder in order to investigate the effect of radiation treatment on the color strength of dyed fabric. The color fastness to light, rubbing- and washing showed that gamma irradiation has improved the dyeing characteristics from fair to good [88]. Eucalyptus (*Eucalyptus camaldulensis*) bark powder (un-irradiated and irradiated) has also been used as natural colorant for dyeing un-irradiated and irradiated cotton fabric using different absorbed doses of gamma irradiation to study the effect of radiation treatment on the color strength of dyed fabrics and found that gamma irradiation has a potential to improve the fastness properties of dyed cotton [89]. Recently, investigations have been carried out in spectraflash, showed that gamma ray treatment of 30 kGy capacity was found optimum dose onto fabric’s surface modification. Lutein as a colorant extracted from marigold was observed to have ability for improvement in dye uptake, color strength and fastness criteria, significantly [90, 91].

**Enzymatic Processing**: Enzymatic processing also has been used as a sustainable and eco-friendly method for textile coloration and functionalization [92]. Three enzymes named protease-amylase, diasterase and lipase were complexed with tannic acid as a pretreatment on cotton and silk, and dyed with natural dyes to evaluate effect of enzymatic treatment on color characteristics. The enzymatic treatment was found to give cotton and silk fabrics rapid dye adsorption kinetics and total higher adsorption than untreated samples [93]. Advanced technologies and methods of recent times for dyeing are accelerating the development in textile industry owing to the sustainability and environment friendly nature of them.

**Fig. 5:**
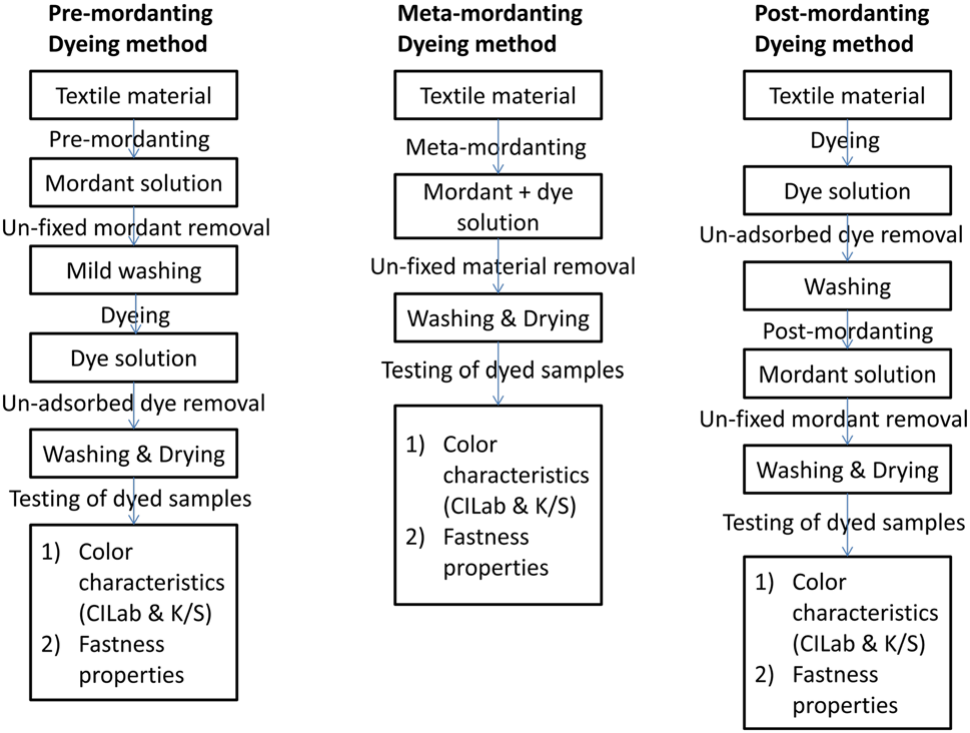
Flow chart for dyeing methods with different mordanting techniques

**Fig. 6:**
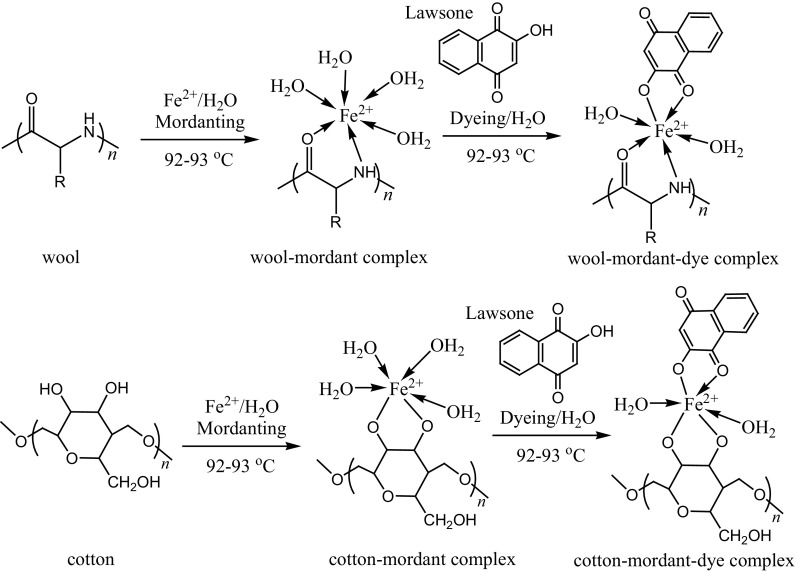
Plausible interaction of fibre-mordant-dye complex (For simplification, lawsone molecule is taken as dye)

Representative schemes shown in Figs. 5 and 6 describe the flow chart for dyeing methods with different mordanting techniques and plausible interaction of fibre-mordant-dye complex, respectively.

### Sustainability

In 1856, William Henry Perkin, while experimenting with coal tar in the hope of finding an artificial quinine as a cure for malaria, discovered the first violet synthetic dyestuff which he called *Mauve*. After the advent of synthetic dyes and their immediate acceptability throughout the world, the use of natural dyes in textile coloration industries slowly became a thing of the past [33, 40]. Extraction of the colorant from biomass depends on the extraction technique employed and, it can be noted that the full range of colors might not yet be available for further application. Considerable weak discernible residues associated with the use of bio-colorants are: reproducibility, cost efficiency, inadequate degree of fixation, and low color fastness properties [59, 93, 94]. These drawbacks of natural dyes can be overcome with the use of appropriate mordants which are permissible up to some levels for textile dyeing [13, 95]. However, due to environmental concerns and eco-protection has created the revival interest of R&D in the use of bio-colorants worldwide. Environmental awareness and pollution concerns implied ban on benzidine and azo dyes which produce any one of the 22 amines related with their carcinogenicity (Table 3).

**Table 3:** ETAD banned aromatic amines with CAS Numbers [94, 152]

Aromatic amines	CAS numbers
4-Aminoazobenzene	60-09-3
*o*-Anisidine	90-04-0
2-Naphthylamine	91-59-8
3,3′-Dichlorobenzidine	91-94-1
4-Aminodiphenyl	92-67-1
Benzidine	92-87-5
*o*-Toluidine	95-53-4
4-Chloro-*o*-toluidine	95-69-2
4-Methyl-1,3-phenylenediamine	95-80-7
*o*-Aminoazotoluene	97-56-3
5-Nitro-*o*-toluidine	99-55-8
4,4′-Methylene-bis-(2-chloraniline)	101-14-4
4,4′-Methylenedianiline	101-77-9
4,4′-Oxydianiline	101-80-4
*p*-Chloraniline	106-47-8
3,3′-Dimethoxybenzidine	119-90-4
3,3′-Dimethylbenzidine	119-93-7
*p*-Cresidine	120-71-8
2,4,5-Trimethylaniline	137-17-7
4,4′-Thiodianiline	139-65-1
4-methoxy-*m*-phenylenediamine	615-05-4
4,4′-Methylenedi-*o*-toluidine	838-88-0

Further, the improvement in color fastness abilities to textile materials can be made using metallic salts under eco-limits. For example, alum, iron mordant were accepted for their improved fastness properties and broadening the color range. Currently, the studies on plant extracts as novel alternate to conventional mordants has proved more sustainability in natural dyeing system. Although metallic mordants are used to enhance the affinity of natural dyes to textile fibers, they generate wastewater containing residual toxic metal ions which leave negative impacts on the environment and, cause severe health-related problems and allergic responses [96, 97]. Consequently, researchers searched for cleaner and greener substitutes from biomes and, green alternatives having high tannin and/or metal hyper-accumulating contents have been employed [3, 13, 98–100].

To adapt the use of bio-resourced materials for textile coloration and finishing, they should be reach the technical, eco-preservation, economic and ecological requirements of the twenty first century by which, equity and sustainability might be considered. Also, the unused residues by the process of natural dyeing can be returned to agriculture for composting or gasification for biogas production. Dyes from natural origin are believed healthier over synthetic ones, but due to lower substantive nature, durability and narrow shade range on textiles of them need further advancement in the application of bio-colorants for coloration and finishing of textile materials [13, 68]. Thus, from the point of environmental safety, bio-colorants serve as promising and sustainable alternative to their synthetic counterparts.

## Adsorption and Kinetic Aspects

Adsorption isotherms, thermodynamic and kinetic studies of dyeing are very much important to study the mechanism of dyeing with colorants on textiles. Some literature regarding these studies with synthetic as well as natural colorant’s application on textiles and textile materials available can be very much helpful to investigate the bonding and the dyeing parameters. These types of studies were popular for adsorption of pollutants from water bodies by various adsorbents for waste water treatment purposes [101]. But, to advance the conventional dyeing to developed technology with better results with the use of minimum sources, these studies are gaining popularity in textile dyeing.

Sun and Tang [101] studied the adsorption properties of honeysuckle aqueous extract’s application for dyeing wool. Kinetic equations such as pseudo-first-order, pseudo-second-order, Elvoich, and intra-particle diffusion equations were employed and pseudo-second-order was found best fit for the adsorption data. Freundlich, Langmuir, Redlich-Peterson, and Langmuir-Nernst isotherm models were studied for their fitting to the adsorption data and Redlich-Peterson, and Langmuir-Nernst isotherm models were found best fitted with the data. Pseudo-second-order kinetic equation fitting of honeysuckle [102] onto wool justified the adsorption mechanism as a chemisorption process, involving the valency forces through the sharing or exchange of electrons between adsorbent and adsorbate as covalent force and ion exchange, also found in case of sodium copper chlorophyllin on silk [103], lac dye on wool and silk [104], and indigo carmine onto silk [105]. Various schemes of adsorbed dyes on textile materials were given according to the adsorption and kinetic studies for simplification of understanding of the chemical interactions between dye and textiles. Langmuir adsorption isotherm is considered as most common for dyeing processes, defined mainly for the monolayer and homogenous adsorption on the surfaces.

Adsorption isotherms to study dye adsorption on textiles:

### Langmuir Adsorption Isotherm Model

Langmuir adsorption isotherm model assumes the monolayer, homogenous adsorption over the surface and kinetic modelling [103–105]. Adsorption occurs on definite localised sites on surface and the layer adsorbed is of one molecular thickness. In this isotherm derivation there is molecules adsorbed are considered of same sorption energies and affinity for adsorption. Once a layer occupied with molecules, adsorption process saturates and the graphically a plateau obtained [106].

A mathematical linear equation represents the Langmuir adsorption isotherm model is [94]:

$$\frac{1}{{C_{f} }} = \frac{1}{{S_{c} }} + \frac{1}{{S_{c} K_{l} C_{s} }},$$

where *C*_*f*_ and *C*_*s*_ are the amount of dye adsorbed per gram of wool fibre and dye concentration in dye bath at equilibrium, respectively. *S*_*c*_ is the maximum dye adsorbed per unit weight of wool fibre for complete monolayer adsorption. K_L_ is Langmuir constant related to affinity of binding sites.

The essential characteristics of Langmuir isotherm can be expressed in terms of the dimensionless constant separation factor for equilibrium parameter, R_L_, defined as follows:

$$R_{L} = \frac{1}{{1 + K_{L} C_{O} }},$$

where C_0_ is the initial dye concentration (mg L^−1^) and K_L_ is Langmuir constant.

### Freundlich Adsorption Isotherm Model

Freundlich adsorption isotherm model is the earliest known adsorption model for multilayer adsorption describes the non-ideal and reversible adsorption. This model can be applied to the heterogeneous surfaces. According to this model, sites with higher binding energy occupied first and others thereafter. Generally not found fitted for natural dyes adsorption on textile materials [107].

Mathematically represented by equation for linear form:

$$\ln C_{f} = \ln K_{f} + 1/n\ln C_{s} ,$$

where *C*_*f*_ and *C*_*s*_ are the amount of dye adsorbed per gram of wool fibre and dye concentration in dye bath at equilibrium respectively. *K*_*f*_ is the Freundlich adsorption constant and *n* is that of the adsorption intensity.

### Temkin Adsorption Isotherm Model

Temkin isotherm is also an early model describes mainly the adsorption of hydrogen onto platinum electrodes within acidic solutions. This isotherm contains a factor that explicitly taking into account of adsorbent-adsorbate interactions. The model assumes that heat of adsorption (function of temperature) of all molecules in the layer would decrease linearly rather than logarithmic with coverage by ignoring the extremely low and large value of concentrations. Its derivation is characterized by a uniform distribution of binding energies and Temkin equation is excellent for predicting the gas phase equilibrium, but complex adsorption systems including the liquid-phase adsorption isotherms are usually not represented by this model [108].

$$q_{e} = \frac{RT}{{b_{T} }}\ln A_{T} + \left( {\frac{RT}{{b_{T} }}} \right)\ln C_{e}$$

where *A*_*T*_ is Temkin isotherm equilibrium binding constant, *b*_*T*_ is Temkin isotherm constant, R and T are universal gas constant and Temperature respectively.

### Hill Isotherm Model

Hill equation, originated from the NICA model, was proposed to describe the binding of different species onto homogeneous substrates. The model assumes that adsorption is a cooperative phenomenon, with the ligand binding ability at one site on the macromolecule, may influence different binding sites on the same macromolecule [109, 110].

Linear equation representation of Hill isotherm model is:

$$\log \left( {\frac{{q_{e} }}{{q_{{s_{H} }} - q_{e} }}} \right) = n_{H} \log \left( {C_{e} } \right) - \log \left( {K_{D} } \right),$$

where *C*_*e*_ equilibrium concentration (mg/L), *q*_*e*_ amount of adsorbate in the adsorbent at equilibrium (mg/g), $q_{{s_{H} }}$; Hill isotherm maximum uptake saturation (mg/L), *n*_*H*_ Hill cooperativity coefficient of the binding interaction, and *K*_*D*_ Hill constant.

### Redlich–Peterson Isotherm Model

Redlich–Peterson isotherm is a combined isotherm of both Langmuir and Freundlich isotherms, which incorporate three parameters into an empirical equation. The model is evaluated to represent adsorption equilibrium over a wide concentration range, that can be applied either in homogeneous or heterogeneous systems due to its versatility. In the limit, it approaches Freundlich isotherm model at high concentration and is in accordance with the low concentration limit of the ideal Langmuir condition [111, 112].

$$\ln \left( {K_{R} \frac{{C_{e} }}{{q_{e} }} - 1} \right) = g\ln \left( {C_{e} } \right) + \ln \left( {a_{R} } \right),$$

where *a*_*R*_ Redlich–Peterson isotherm constant (1/mg), *K*_*R*_ Redlich–Peterson isotherm constant (L/g), *C*_*e*_ equilibrium concentration (mg/L), *q*_*e*_ amount of adsorbate in the adsorbent at equilibrium (mg/g), and *g* Redlich–Peterson isotherm exponent.

Common kinetic models used for sorption studies [113, 114] are discussed below:

### Pseudo-First-Order

Lagergren suggested a rate equation for the sorption of solutes from a liquid solution. This pseudo-first-order rate equation is expressed as:

$$\frac{{dq_{t} }}{dt} = K_{1} \left( {q_{e} - q_{t} } \right),$$

where *q*_*e*_ and *q*_*t*_ are the sorption capacity at equilibrium and at time *t*, respectively, and *K*_1_ is the rate constant of pseudo-first order sorption.

Integrated equation for pseudo-first-order kinetics is:

$$\log \left( {q_{e} - q_{t} } \right) = \log \left( {q_{e} } \right) - \frac{{K_{1} }}{2.303}t,$$

$\log \left( {q_{e} - q_{t} } \right)$ verses *t* straight line plot gives fitting of pseudo-first-order kinetics for adsorption of dye on to textile surfaces.

### Pseudo-Second-Order

Pseudo-second-order kinetic model fitting justifies the chemisorption process in textile dyeing, with the adsorption followed by chemical forces such as ionic bonding, coordinate bonding and H-bonding etc. Pseudo-second-order kinetic model rate equation can be expressed as:

$$\frac{{dq_{t} }}{dt} = K\left( {q_{e} - q_{t} } \right)^{2} ,$$

where *q*_*e*_ and *q*_*t*_ are the sorption capacity at equilibrium and at time *t*, respectively and *K* is the rate constant of pseudo-second order adsorption kinetics.

Integrated rate equation for of pseudo-second order adsorption kinetics is:

$$\frac{1}{{\left( {q_{e} - q_{t} } \right)}} = \frac{1}{{q_{e} }} + Kt.$$

And can also be solved further and written as:

$$\frac{t}{{q_{t} }} = \frac{1}{{Kq_{e}^{2} }} + \frac{1}{{q_{e} }}t,$$

Straight line fitting in the graph of $${\raise0.7ex\hbox{$t$} \!\mathord{\left/ {\vphantom {t {q_{t} }}}\right.\kern-0pt} \!\lower0.7ex\hbox{${q_{t} }$}}$$ verses *t* gives better correlation of pseudo-second order adsorption kinetics of dye adsorption.

## Functional Applications

### Antimicrobial Finished Textiles

All textiles provide a growing environment for these micro-organisms. Natural fibres, such as cotton and wool, are especially susceptible to microbial growth and even dust mites because they retain oxygen, water and nutrients. Micro-organisms can embed themselves in clothes in a closet, curtains, carpets, bed, bath and kitchen linens, even pillows and mattresses. Many bacteria also grow on the skin while dust mites live on shed, human skin cells that have been deposited on items such as sheets, towels, and clothing. Like a house, a hospital contains an immense amount of textiles with the added threat of high transmission of microorganism.

Antimicrobial agents from both synthetic and natural origin were applied to get rid of these microorganisms. Due to eco-friendly nature of natural origin agents, are to be more favoured in the textile finishing. In past, natural dyes were applied to textiles for simultaneous coloration and antimicrobial finishing successfully. An attempt to examine the effect of *Rheum emodi* L. as dye and its dyed wool yarns activity against two bacterial (*Escherichia coli* and *Staphylococcus aureus*) and two fungal (*Candida albicans* and *Candida tropicalis*) species was studied and resulted into successful antimicrobial finishing of wool fibres [59]. Evaluation of antimicrobial activity of catechu in solution and % microbial reduction of dyed wool samples against *Escherichia coli* MTCC 443, *Staphylococcus aureus* MTCC 902, *Candida albicans* ATCC 10261 and *Candida tropicalis* ATCC 750, by using micro-broth dilution method, disc diffusion assay and growth curve studies were studied with Haemolytic activity on human erythrocytes to exclude possibility of further associated cytotoxicity. Observed antimicrobial characteristics and negligible cytotoxicity of catechu indicated the dye as a promising antimicrobial agent for developing bioactive textile materials and clothing [65, 115, 116].

The inherent properties of the textile fibres provide room for the growth of micro-organisms. Besides, the structure of the substrates and the chemical processes may induce the growth of microbes. Humid and warm environment still aggravate the problems. Infestation by microbes cause cross infection by pathogens and development odor where the fabric is worn next to the skin [69]. Experimentation of synthetic/natural materials with antimicrobial finishing opened many doors for scientists. As knowledge of functional finishes and manmade fibres evolved, so did society’s view on health and safety. With this increase in health awareness, many people focused their attention on educating and protecting themselves against harmful pathogens. It soon became more important for antimicrobial finished textiles to protect the wearer from bacteria than it was to simply protect the garment from fibre degradation.

All textiles provide a growing environment for these micro-organisms. Natural fibres, such as cotton and wool, are especially susceptible to microbial growth and even dust mites because they retain oxygen, water and nutrients. Micro-organisms can embed themselves in clothes in a closet, curtains, carpets, bed, bath and kitchen linens, even pillows and mattresses. Many bacteria also grow on the skin while dust mites live on shed, human skin cells that have been deposited on items such as sheets, towels, and clothing. Like a house, a hospital contains an immense amount of textiles with the added threat of high volumes of traffic. Because of the constant flow of people, especially those with infectious diseases, many researchers have focused on creating finishes specifically for hospital use. Both patients and employees are at risk for cross transmission of diseases and other health issues. The majority of these microorganisms are passed from person to person by various textiles. The increasing rate of drug-resistant bacteria only heightens the importance of finding safe and durable antimicrobial finishes. Several elements and natural compounds have inherent antimicrobial properties. Heavy metals and metallic compounds hold a large portion of the market for antimicrobial textiles. Cadmium, silver, copper, and mercury are all effective antimicrobial agents. Metal based finishes are fairly durable to repeated laundering making them appropriate for use as a reusable finish. Several natural, non-metallic, antimicrobial finishes exist. One of these natural antimicrobial finishes, Chitosan, is the deacetylated form of Chitin which is a main component in crustacean shells. Chitosan has been shown to be effective against both gram-positive and gram-negative bacteria [117–119]. Researchers have responded to problems like this by experimenting with the currently available finishes available. Many antimicrobial textiles are treated with combinations of bioactive substances to enhance the antimicrobial efficacy of the finishes and counter act the negative aspects of the treatments. By combining finishes, the occurrence of drug resistant strains forming from the finish is decreased. Another trend in experimentation with antimicrobial finishes consists of adding antimicrobial agents to synthetic fibres during the spinning process.

Although known for a long time for dyeing as well as medicinal properties, the structures and protective properties of natural dyes have been recognized only in the recent past. Many of the plants used for dye extraction are classified as medicinal, and some of these have recently been shown to possess remarkable antimicrobial activity. Some common natural dyes have been showed antimicrobial activity such as curcumin from turmeric, naphthoquinones such as lawsone from *Lawsonia inermis*, juglone from walnut, lapachol from taigu, catechin from *Acacia catechu*, several anthraquinones such as *Rubia tinctorum*, *Rubia cordifolia*, *Rheum emodi*. *Punica granatum* and *Quercus infectoria* natural dyes are reported as potent antimicrobial agents owing to the presence of a large amount of bioactive phytochemicals [34, 120–123].

Since, the synthetic antimicrobial agents are associated with the release of enormous amount of hazardous chemicals to the environment which, are cause of many skin disorders and related diseases, during their processing and application. To minimize the risks associated with the application of synthetic antimicrobial agents, there is a great demand for antimicrobial textiles based on non-toxic and eco-friendly bioactive compounds. Due to the relatively lower incidence of adverse reactions of natural products in comparison with synthetic pharmaceuticals, they can be exploited as an attractive and eco-friendly alternative for textile applications [3, 13, 124, 125]. Although there are many natural antimicrobial agents, may significantly reduce the risk of infections especially when they are used in close contact with the patients or in the immediate and non-immediate surroundings. Natural bioactive compounds (natural dyes/pigments) have been reported as significant antimicrobial agents for textile finishing in eco-friendly dyeing.

### UV Protective Textiles

Ultraviolet rays, a low fraction of solar spectrum influences all living organisms and their metabolisms. These Ultraviolet rays exposure can cause effects from tanning to skin cancers. Sunscreen lotions and clothing provide protection from the harmful effects of ultraviolet radiations. Alterations in the construction parameters of fabrics with appropriate light absorbers and suitable finishing methods can be employed as UV protection fabrics.

Three natural yellow dyes, namely *Rheum emodi*, *Gardenia yellow* and *curcumin*, were successfully applied for simultaneous dyeing and functionalization of silk to get UV protection abilities for textiles [126]. Dye extracted from the leaves of eucalyptus and applied to wool fabric by using two padding techniques, namely the pad-batch and pad-dry techniques under different conditions and it was observed that with an increase in the dye concentration, the ultraviolet protection factor (UPF) values ranged between very good and excellent for wool fabric [127]. UV-protection properties of chlorogenic acid, main ingredient of water-extract from honeysuckle, on wool were studied. The honeysuckle extract showed good UV transmittance in the range of UVA and UVB of wool treated with honeysuckle extract and thus extract of honeysuckle may be developed as a natural UV-absorbing agent applied to wool finishing [102]. Natural plant colorants madder (*Rubia tinctorum*) and indigo (*Indigofera tinctoria*) and the natural colorant of insect origin cochineal (*Dactylopius coccus*) were applied on cotton fabrics and tested for UV protection abilities, among them indigo was observed as having higher UPF values [4, 128].

### Deodorizing finishing

As far as, new generation is concerned about health and hygiene in recent time, there are more advancement to improve the performance of textiles with respect to odour with antimicrobial and UV protection properties. Grown bacterial colonies or waste released from human body are the main causes for odour in garments. To meet the consumer’s mature demand for hygienic clothing, extensive significant work has been published regarding the deodorizing property of textiles achieved with the application of natural colorants. The deodorizing performance of fabrics dyed with natural colorant extracts was comparatively studied and deodorizing efficiency of pomegranate was dominated among gardenia, *Cassia tora*. L., coffee sludge and pomegranate rind [129]. Gallnut dyed fabrics showed a better deodorizing function against ammonia, trimethyl amine and acetaldehyde, compared to the un-dyed fabrics. Also the dyed fabrics showed an excellent antibacterial activity against *Staphylococcus aureus* and *Klebsiella pneumonia* [130]. Cotton, silk and wool fabrics dyed with pomegranate (*Punica granatum*) extract by Young-Hee Lee and co-workers for deodorizing functionalization and found excellent results in range of 99% [131]. Cotton fabrics were dyed with C. I. Direct Blue 200, a copper complex direct dye, and pre and post-mordanted with Cu(II) sulfate for deodorization of ethyl mercaptan. According to the results, all the deodorization effects plotted against the copper ion uptakes were found to increase quadratically with the copper ion uptake [132]. Thus, natural as well as synthetic dyes can be utilised for deodorizing functionalization of textiles by following proper protocol (optimized) of dyeing.

### Moth Resistant and Insect Repellent Textiles

Wool and other hair fibres used for producing carpets, blanket and shawl etc. due to having properties like warmth, softness and flame retardancy. Specially, wool-based materials due to its protein content are prone to attack of moth and other insects. Moth is an insect and its larvae eat the protein present in wool. Cloths moth (*Tineola bisselliella*) and carpet beetle (*Anthrenus verbasci*) are common moths attacking the wool materials. DDT (Dichlorodiphenyltrichloroethane), permethrin, permethrin/hexahydro pyrimidine derivative, cyhalothrin, etc. are some of the chemicals used as antimoth finishing agents. Nano TiO_2_ particles were also utilized as an antifeeding compound on wool fabric against larvae of the carpet beetle, *Anthrenus verbasci*, feeds on protein fibers [133]. All the chemicals used for antimoth finishing are associated with ecological disturbance and so, natural colorants may be perfect substitutes for them. Shakyawar et al. screened saffron flower waste, onion skin, henna, myrobolan, silver oak leaf, madder, walnut, dholkanali and yellow root natural dye sources for antimoth finishing and found best results for silver oak leaves, walnut husk and pomegranate rind [134]. Natural dyes cochineal, madder, and walnut (quinines) and chestnut, fustic, indigo, and logwood (flavonoids) were applied on wool and tested for antimoth properties against black carpet beetles. All of the dyes, except indigo, increased the insect resistance of the wool fabric but flavonoid dyes were not so effective in enhancing insect resistance. Metallic mordants were found not having significant effect on insect resistance with all natural dyes used. The anthraquinone dyes including cochineal, madder, and walnut were found to be quite effective in protecting wool fabric against black carpet beetles [135].

### Food Coloration

Foods are typically made colored so that they seem to be more appealing, appetizing and to match the flavors added. In the present era of eco-safety and eco-preservation, growing worldwide concern for food quality and safety, have been brought in by national governments. A particular country has its own basic regulations and acceptable standards for synthetic colors as additives for food and these can be up and down to other country. Natural originated colors are approved over the years and, scientific communities have devoted more attention in the development of greener substitutes, as they are generally more internationally accepted [136, 137]. The use of bio-colorants in food coloration have gaining popularity among food manufacturers as well as consumers in determining the acceptability of processed food and, there has been seeking advancements of new natural colorants for use in food industry in the continuing replacement of synthetic food dyes, which are not found in nature and, often are azo-dyes that being unsafe, created a big challenge for scientific community. Currently, the European Union has authorized approximately 43 colorants as food additives, out of which approximately 30 color additives are approved in the United States [138–140].

Prominent academicians and R&D researchers all over the world, have keen to experiment and expand this interesting palette of natural food color choices to give distinguished look and quality to an array in bio-based food coloration. The sources of natural bio-colorants for food coloring and styling primarily are, certain species of plants, animals and microorganisms. Considerable increment of public awareness about the use of synthetic color additives for food products has augmented in the use of natural food colorants. In 1991, Japanese food legislation on the statement of natural food additives on labels was enforced due to the several impacts on public health and, call to reliable methods, especially natural food colorants, in food products. Systematic researches make available bio-colorants such as, carotenoids [141, 142], anthocyanins [143, 144], betalains [145, 146], chlorophylls [147, 148], tannins [149], quinones [115, 150], biliproteins [151] etc. All, they have different auxochromic and chromophoric groups which directly or indirectly alters to produce different hues ranging from green through yellow, orange, red, blue, and violet, depending on the source of colorant [3, 116, 152]. Consequently, a great upsurge has been seen in biotechnological production of food grade pigments Colorants from microbial world such as fungi, bacteria and microalgae etc. are quite common in nature. Among the molecules produced are carotenoids, melanins, flavins, quinones and more specifically monascins, violacein, phycocyanin or indigo. Synthetic colors in limited quantities are permitted in various types of foods: fruit and vegetable products, hard-boiled confectioneries, bakery foods, instant foods, traditional Indian sweetmeats and other dairy products. However, synthetic colors are being replaced by natural colors in view of the health benefits as well as increased public awareness towards eco-preservation.

## Future Prospective and Conclusion

In the present scenario, the growing concerns among the communities globally against the use of azo and benzidine synthetic dyes due to their carcinogenic, non-biodegradable nature and hazardous effects on environment and human health, re-established the needs of natural dyes to human society in terms of packaging and daily use products [41, 94, 153]. With increase in awareness for eco-friendly materials from sustainable resources, natural dyes attracted researchers in traditional and diversified applications to develop effective eco-friendly and cleaner process technologies [3]. Natural dyeing is gradually making its way in the global market and the production of naturally dyed eco-friendly textiles itself is a boon to save the environment from hazardous synthetic dyes. However, the color derived from raw plant materials is known to be very sensitive to the food processing conditions but, in general, eco-friendly criterion paid safely to reconsideration of technological parameters, with more attention to their effects on color stability, is therefore advisable and could be promisible alternate to artificial colorants. Furthermore, the fast moving inexpensive synthetic dyes stand as a big question before natural dyers. But, the non-toxic, non-carcinogenic, bio-degradable and eco-friendly characteristics of naturally derived colorants made its own way to reach the hearts of conscious consumers for healthy lifestyle, and can be achieved on a higher cost [1, 93]. Hence, the applications of bio-colorants to textile substrates shall be helpful to entrepreneurs to take up this venture which have good potential and bright future in a number of applied sectors: leather, textiles and clothings, cosmetics, food, pharmaceutical, and paint industries etc.

Naturally derived pigments are available in nature with various hues and tones, currently exploited for the coloring of textile and food materials, and other several other biomedical applications. New sources of biomass biased pigments need to be available in sufficient quantities for stability during processing and storing for large-scale cultivation, industrial extraction, formulations, harvesting and storage and, application of biotechnological tools including cell and tissue cultures, genetic engineering, promoted by experts as a replacement for conventional growing techniques. Modem consumer’s demand for novel eco-materials tend to the expansion in bio-colorant list towards forthcoming future. Evenly, recent advances have been performed in the development and applications of natural colorants covering different aspects such as identification of new sources, formulations, extraction, purification and stability techniques. In spite of enthusiastic studies discussed the data for the socio-economic viability of natural dye production and applications at commercial scale for sustainable utilization of bio-resources, related to hygiene and eco-safety which have a great future scope for the discovery of relatively better and more stable natural pigments that may have wider industrial applications. More experimental implementations should be focused to adopt novel technologies for making natural colorants as a compatible as well as eco-safe alternative with synthetic colorants in different spheres of our life to make a greener world.
